# Clinical Outcomes for Breast Cancer Patients Undergoing Mastectomy and Reconstruction with Use of DermACELL, a Sterile, Room Temperature Acellular Dermal Matrix

**DOI:** 10.1155/2014/704323

**Published:** 2014-03-11

**Authors:** Christopher Vashi

**Affiliations:** ^1^The Plastic Surgery Group, Chapel Place II, 340 Thomas More Parkway, Crestview Hills, KY 41017, USA; ^2^The Plastic Surgery Group of Greater Cincinnati, 4850 Red Bank Expressway, Cincinnati, OH 45227, USA

## Abstract

*Background*. Decellularized human skin has been used in a variety of medical applications, primarily involving soft tissue reconstruction, wound healing, and tendon augmentation. Theoretically, decellularization removes potentially immunogenic material and provides a clean scaffold for cellular and vascular in growth. The use of acellular dermal matrix in two-stage postmastectomy breast reconstruction is described. *Methods*. Ten consecutive breast cancer patients were treated with mastectomies and immediate reconstruction from August to November 2011. There were 8 bilateral and 1 unilateral mastectomies for a total of 17 breasts, with one exclusion for chronic tobacco use. Reconstruction included the use of a new 6 × 16 cm sterile, room temperature acellular dermal matrix patch (DermACELL) soaked in a cefazolin bath. *Results*. Of the 17 breasts, 15 reconstructions were completed; 14 of them with expander to implant sequence and acellular dermal matrix. Histological analysis of biopsies obtained during trimming of the matrix at the second stage appeared nonremarkable with evidence of normal healing, cellularity, and vascular infiltration. *Conclusion*. Postoperative observations showed that this cellular dermal matrix appears to be an appropriate adjunct to reconstruction with expanders. This acellular dermal matrix appeared to work well with all patients, even those receiving postoperative chemotherapy, postoperative radiation, prednisone, or warfarin sodium.

## 1. Introduction

For breast cancer patients, the use of expanders and/or implants is the most common method of breast reconstruction following mastectomy [1]. This typically involves a two-stage process where tissue expanders are placed postmastectomy and filled gradually for a period of several months. Once the desired expander volume is reached, the second reconstructive stage involves replacing the expanders with silicone implants. Another reconstruction option is the use of autologous tissue from a separate patient site to supply needed skin for wound closure at the mastectomy site. However, the cosmetic issues of this method remain negative for patients due to scarring and differences in skin coloration between the autologous and surrounding tissues. Donor site morbidity also remains a concern for many patients. When postmastectomy implantation is feasible and desired, structural support of the breast can provide ideal shape and implant positioning. Such support can be accomplished by introducing biocompatible mesh, but there have been some complications observed with this procedure [2].

As an alternative, acellular dermal matrices (ADMs) are produced by the removal of the epidermal layer from thin slices of skin, leaving the dermal layer and extracellular matrix followed by a decellularization process. The removal of donor cellular material including major histocompatibility complex (MHC) proteins is performed to theoretically minimize immunological response in ADM recipients [3] and promote revascularization and cellular infiltration [4], thus yielding clinically promising materials for soft tissue reconstruction, wound healing, and tendon augmentation [5–24]. However, there exists a nascent complication rate with ADMs in breast reconstruction procedures, including seromas and reports of “red breast syndrome,” an apparent inflammatory response to residual components in the ADM [16, 25–27]. A new ADM, DermACELL, (here referred to as D-ADM) is manufactured using a proprietary decellularization process [28] that removes at least 97% of nucleic acid material, is not freeze-dried, and is provided hydrated with a Sterility Assurance Level (SAL) of 10^−6^. This unique process yields a material that has demonstrated rapid* in vivo* cellular infiltration and vascularization [4] with both properties being advantageous in healing [29]. At the time of this writing, there were no known published reports of the use of this material in breast reconstruction. Here, we report the postmastectomy outcome of D-ADM used in two-stage breast reconstructions.

## 2. Materials and Methods

### 2.1. Study Overview

Ten consecutive female breast cancer patients between the ages of 28 and 60 years old were scheduled to undergo mastectomies from August to November 2011. All eligible patients were included with criteria for exclusion being tobacco use (smoking) or a known planned course of postoperative radiation after mastectomy. One patient was excluded prior to beginning the study under the criteria of smoking and two patients did have previously unplanned radiation treatments following ADM implantation due to unanticipated laboratory results and were still included in the series. Procedures for the 9 remaining patients included 8 bilateral mastectomies and 1 unilateral mastectomy for a total of 17 breasts in the study. The final filling volumes of their tissue expanders ranged between 450 and 800 cc. Eight patients totaling 14 breasts advanced to the 2nd stage operation which involved removing the tissue expanders to be replaced with silicone implants. One of the eight patients lost the right expander, presumably due to smoking. Subsequently, this patient received an autologous Transverse Rectus Abdominis Myocutaneous (TRAM) flap on the right and completed the expander to implant exchange on the left. The ninth patient opted for bilateral expander removal after metastatic disease was diagnosed.

### 2.2. Clinical Procedure and Implant Description

**Figure 1:**
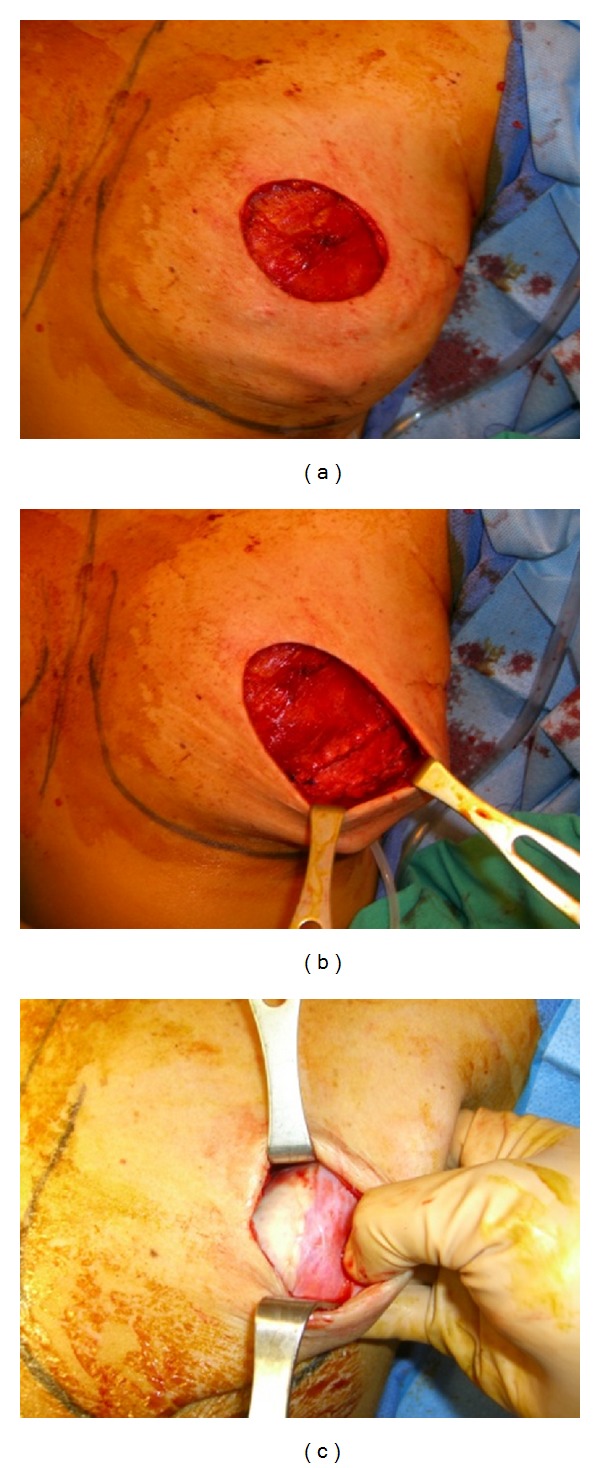
(a) Showing placement of expander. (b) Showing D-ADM and intraoperative expansion. (c) Showing D-ADM incorporation at second stage reconstruction.

The mastectomies were performed by a total of 4 general surgeons. The D-ADM (DermACELL, LifeNet Health, Virginia Beach, VA) is manufactured [28] using a combination of nondenaturing anionic detergent (N-Lauroyl sarcosinate), recombinant endonuclease (Benzonase), and antibiotics (Polymixin B, Vancomycin, and Lincomycin) and then terminally sterilized with a low dosage of gamma irradiation at low temperatures to a SAL of 10^−6^. The material is never freeze-dried and is stored at room temperature, ready to use. The 6 × 16 cm D-ADM patches were soaked in a Cefazolin (ANCEF, GlaxoSmithKline, Philadelphia, PA) bath and split along a hypotenuse. When possible, intraoperative expansion was performed to the point of light tension on closure. Two drains were placed, one in the superior/axilla area and one in the inframammary fold at the D-ADM application site. Expansion began at 3 weeks postop. at a rate of 30–60 cc per week even if drains remained in place. An example of the surgical procedure is shown in Figure 1.

### 2.3. Case Descriptions and Course of Treatment

#### 2.3.1. Patient 1

A 46-year-old patient received bilateral mastectomies on September 9, 2011 and advanced to the 2nd stage on February 28, 2012. She underwent chemotherapy during expansion and developed DVT in the left leg during this period, which was treated with warfarin sodium (Coumadin, Bristol-Myers Squibb Company, New York, NY). Her expanders were filled to the full 550 cc and replaced with 700 cc silicone implants. She has completed nipple areolar reconstruction.

#### 2.3.2. Patient 2

A 55-year-old patient received a unilateral mastectomy of the right breast and required evacuation of a hematoma on the first postoperative day after placement of the D-ADM. At that time, the D-ADM was intact and was not removed. She advanced to the 2nd stage at 6 weeks and to a 3rd stage nipple reconstruction at 16 weeks. She was expanded to 450 cc and received a 500 cc implant. Her areolar micropigmentation was completed several months later.

#### 2.3.3. Patient 3

A 60-year-old patient received bilateral mastectomies on September 26, 2011, was expanded to 450 cc, and advanced to the 2nd stage on January 24, 2012 with 533 cc implants. She opted not to proceed with nipple areolar reconstruction.

#### 2.3.4. Patient 4

A 52-year-old patient received bilateral mastectomies on October 11, 2011, was expanded to 510 cc, and advanced to the 2nd stage at 19 wks with 600 cc implants. She has completed nipple and areolar reconstruction.

#### 2.3.5. Patient 5

A 43-year-old patient received bilateral mastectomies on October 20, 2011. She received unanticipated radiation therapy to the left side and was eventually expanded to 510 cc after radiation. She advanced to the 2nd stage on April 17, 2012 with 600 cc implants. She has completed nipple and areolar reconstruction.

#### 2.3.6. Patient 6

A 28-year-old patient, former smoker, relapsed postoperatively after receiving bilateral mastectomies. She experienced right expander extrusion at 4 wks, and reconstruction was put on hold until smoking cessation. Her left side was fully expanded to 510 cc, and she was reconstructed with a 533 cc implant on the left and a TRAM flap on the right. She has completed nipple reconstruction and remained nicotine free.

#### 2.3.7. Patient 7

A 54-year-old patient received bilateral mastectomies on August 5, 2011. Her drains fell out 4 days postoperatively and she experienced seromas with incisional dehiscence, which required irrigation and drain replacement. She experienced recurrent incisional reopening in the left breast which required left expander removal and replacement after cultures of the excision showed negative gram stains. She was expanded to 800 cc and successfully underwent stage two on March 6, 2012 with 800 cc implants. She has completed nipple and areolar reconstruction.

#### 2.3.8. Patient 8

A 43-year-old patient received bilateral mastectomies on November 7, 2011 and was expanded to 800 cc, and the 2nd stage was completed on February 23, 2012 with 800 cc implants. She has completed nipple and areolar reconstruction.

#### 2.3.9. Patient 9

A 48-year-old patient received bilateral mastectomies on November 4, 2011 and had shown complete response to neoadjuvant chemotherapy by preoperative imaging. She had a positive margin to the chest wall and received postoperative radiation therapy. Despite complete resolution of radiation dermatitis and expansion, she elected to have her expanders removed and abort reconstruction when she was found to have hepatic metastases.

## 3. Results

### 3.1. Clinical Performance

**Figure 2:**
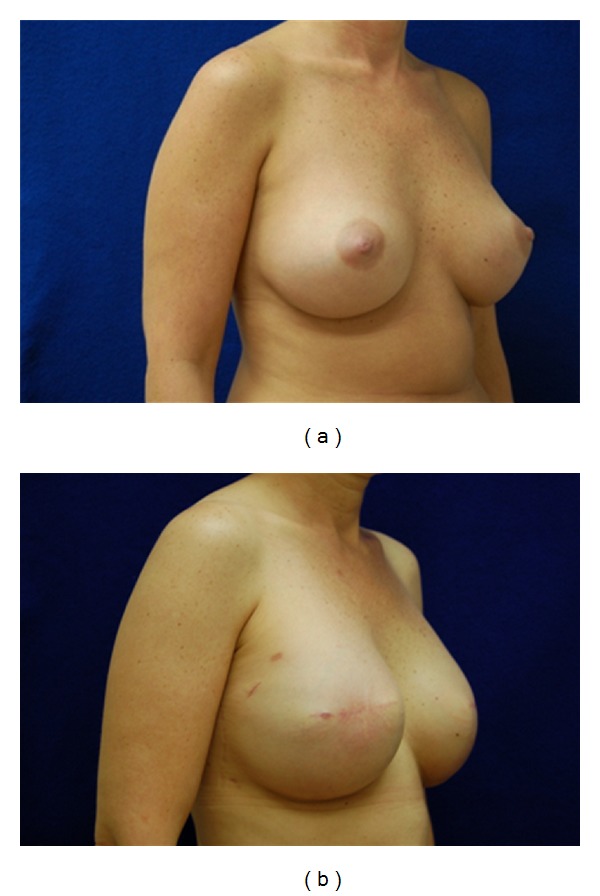
(a) Preoperative before mastectomy. (b) Postoperative after 700 cc silicone implant placed.

**Figure 3:**
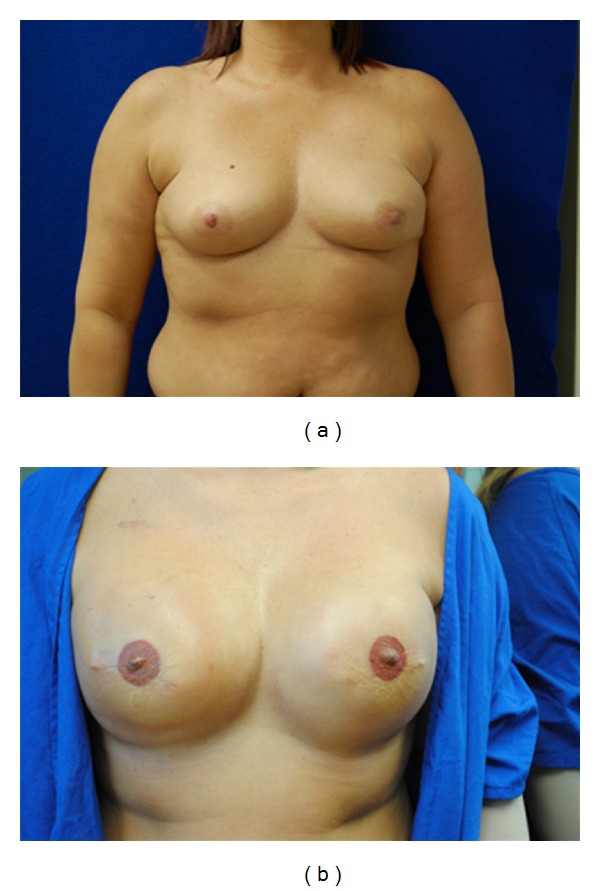
(a) Preoperative before mastectomy. (b) Postoperative after 800 cc silicone implant placed.

**Figure 4:**
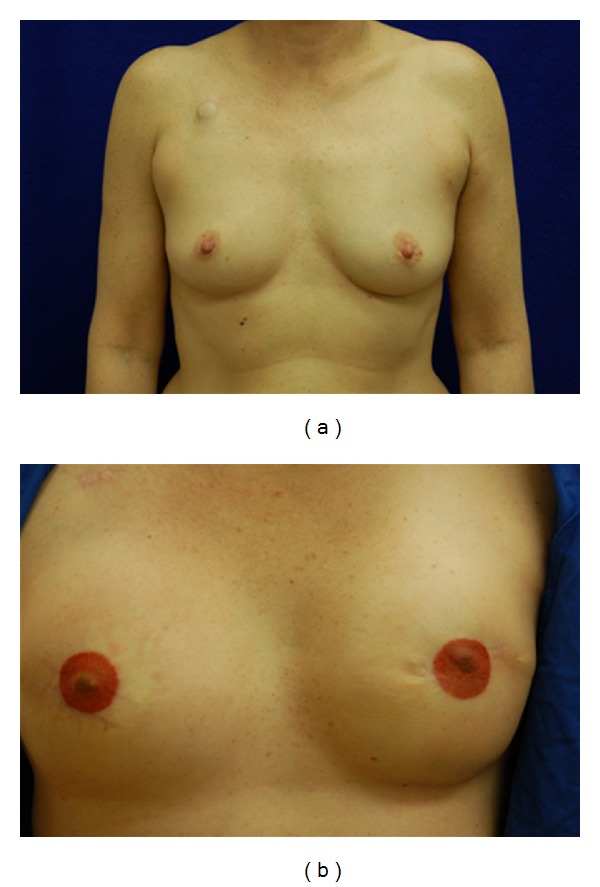
(a) Preoperative before mastectomy. (b) Postoperative after left radiation and reconstruction.

Of the 9 implant patients, most had acceptable results (Table 1). The healthy patients and those with post-ADM placement chemotherapy, hematomas, or warfarin sodium treatment all did well with full recoveries and without further complications, capsule contracture, or need of reoperation. The ADM was 100% adhered and revascularized in all of the patients. Although a demarcation line between the matrix and native capsule was noted (e.g., see Figure 1), the matrix was incorporated at this line. Also, one notable seroma was recorded. Two patients had radiated expanders, and one patient, a smoker, lost a unilateral expander. D-ADM seemed to work well with those patients who received unanticipated postmastectomy radiation treatments. These were scheduled to start three weeks after the mastectomies. Observation, quality, and incorporation of the radiated D-ADM were seen at the second stage of expander to implant exchange and found to be comparable to the nonradiated side in the same patients. Typical patients are shown in Figures 2, 3, and 4. Of particular note was the absence of “red breast syndrome” in all patients, although the limited number of patients in this series precludes any generalizable conclusions.

### 3.2. Histological Analysis

Biopsy specimens were obtained from 8 of the 9 patients and submitted in formalin to Dominion Pathology Laborator (Norfolk, VA) for sectioning and staining. Stains included hematoxylin and eosin (H&E) to assess cellularity and general ultrastructure, immunohistochemical stain CD34, an endothelial cell marker, to assess vascularity, and Verhoeff-Van Gieson (VVG) to assess elastic fibers. Histological assessments were made by dermatopathologists Kevaghn Fair, DO (Dominion Pathology Laboratory) and Antoinette Hood, MD (Eastern Virginia Medical School, Norfolk, VA).

**Figure 5:**
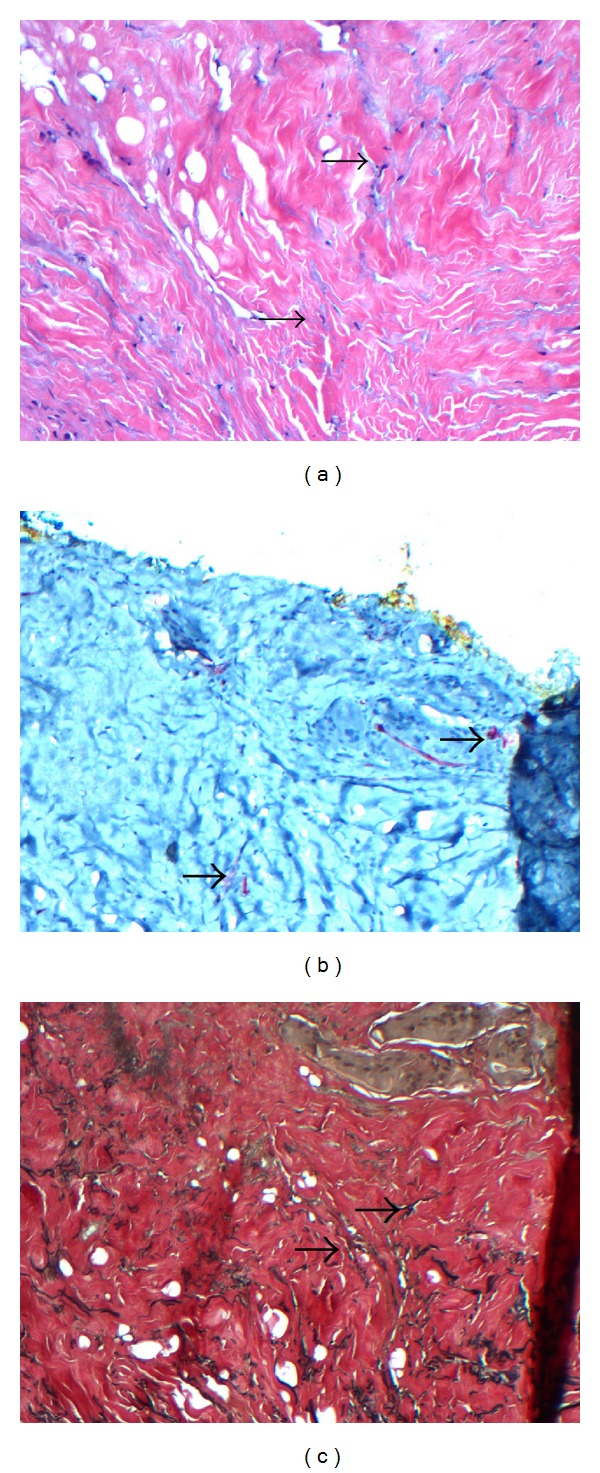
(a) Hematoxylin and eosin staining of biopsy specimen from patient number 1 following 16 weeks* in situ* placement of ADM. Note the intact ultrastructure and also evidence of cellular in growth as apparent fibroblasts (arrows) at 10x magnification. (b) CD34 staining of biopsy from patient number 1 following 16 weeks* in situ* placement of ADM. Evidence of robust vascularization is noted by reddish-brown stains apparently associated with blood vessels (arrows) at 10x magnification. (c) Verhoeff-Van Geisen staining of biopsy from patient number 1 following 16 weeks* in situ* placement of ADM. Note abundance of elastin (arrows) in this 10x magnification.

General histological observations for all biopsied patients included presence of fibroblasts, vasculature, and intact ultrastructure, including elastin. Occasional foreign body response was noted, localized to polarizable material which was present in a regular pattern consistent with suture material. Little inflammation was noted except in conjunction with this foreign body response. In general, the side of the implant facing the expander exhibited pseudocapsule formation as a benign response to the expander material. When observed, the opposite interface between the implant and the host tissue demonstrated some level of tissue integration with minimal inflammation consistent with normal healing. Compared to the host tissue, the implant material appeared more organized with fewer living cells and less vasculature, a finding expected for a stable material slowly being incorporated and remodeled after a few weeks to a few months following surgery when the specimens were collected. Specific histology samples from patient number 1 are shown in Figure 5.

## 4. Discussion

Two-stage breast reconstruction procedures can be facilitated by the use of a sling under the expander for both support and cosmetic benefits. Among many other factors, the choice of this material is key in ensuring a good outcome. Decellularized human skin (ADM) is often used for these procedures. It is hypothesized that certain complications may arise from these materials as a function of successful cellular removal. One of these materials (D-ADM) is validated to remove ≥97% DNA while maintaining structural integrity. Here, we used this material and assessed its performance through patient follow-up and histological analysis ofbiopsies taken upon expander removal. Overall results weregood. One observation of note is that there were no observed drug effects of warfarin sodium and prednisone on the outcome of the procedure. Warfarin sodium use presents a concern for uncontrolled hemorrhaging in these patients, and this was not noted. Prednisone is a corticosteroid drug used in patients with low steroid levels and also used as an anti-inflammatory medication. Clotting and generating an immune response are key biological processes that stimulate wound healing, which are affected to some degree in the patients taking warfarin sodium and prednisone. These patients had no adverse effects postoperatively. One patient exhibited a hematoma that required evacuation on postoperative day one after placement of the D-ADM. At that time, the D-ADM was intact and was not removed. She went on to complete successful expansion. There are several concerns with postoperative hematomas, one of which being the inherent risk of surgery when needed to correct them. Also, the accumulation of blood from hematomas can lead to increased tension on the surgical area causing local infection that prevents proper wound healing [27]. This did not appear to be a factor in the patient with the hematoma in this study. In addition, the absence of “red breast syndrome” in these patients is especially noteworthy. This complication is commonly noted in breast reconstruction procedures using HADM [25, 26]. The factors leading to red breast syndrome are not fully understood, but inflammation in response to the foreign material is thought to be the leading cause. The cause of the absence of red breast syndrome in these patients is unclear, but the complete decellularization of this particular ADM may be a key factor, although the limited sample size prevents firm conclusions. Finally, the patient who relapsed into a previous smoking habit experienced the most complications postoperatively.

## 5. Conclusions

D-ADM appears to be an appropriate adjunct to reconstruction with expanders. D-ADM worked well with patients receiving chemotherapy for further cancer treatment and seemed to work well with those who had received postoperative radiation treatments while the D-ADM was in place. As far as other drug effects on the procedure, there appeared to be none with patients in this study taking warfarin sodium and prednisone as they both responded favorably postoperatively. Additionally, the patient experiencing hematoma responded well with D-ADM despite this complication. Overall, healthy patients had the most favorable results, while those with unhealthy lifestyles, particularly smokers, experienced the most complications.

## Figures and Tables

**Table 1:** Patient overview and results.

Patient number	Age (yrs)	Postop. chemotherapy	Post-ADM implant radiation	Uni- or bilateral	Duration of implant prior to 2nd stage (wks)	Expander size	Implant size	Nipple/Areola reconstruction	Surgical site infection	Seroma
1	46	Yes	No	Bilateral	16	550 cc	700 cc	Yes	No	—
2	55	No	No	R only	6	450 cc	500 cc	Yes	No	—
3	60	No	No	Bilateral	16	450 cc	533 cc	No	No	—
4	52	No	No	Bilateral	19	510 cc	600 cc	Yes	No	—
5	43	No	Yes	Bilateral	16	510 cc	600 cc	Yes	No	—
6	28	No	No	Bilateral	Right side TRAM* and left implant	510 cc	533 cc	Yes	No	—
7	54	No	No	Bilateral	7 weeks	800 cc	800 cc	Yes	Yes	Yes
8	43	No	No	Bilateral	14 weeks	800 cc	800 cc	Yes	No	—
9	48	Neo-adj.	Yes	Bilateral	11/4/2011 stage 1	Aborted	Aborted	Aborted	No	—
10	Excluded due to smoking	—	—	—	—	—	—	—	—	—

*Transverse Rectus Abdominis Myocutaneous.
